# Temperature Dependence of Paramagnetic Species in the Human Brain Tissue: An X‐Band EPR Study

**DOI:** 10.1002/mrm.70222

**Published:** 2025-12-12

**Authors:** André Avanzine, José Henrique Monteiro de Azevedo, Martina Huber, Fábio Seiji Otsuka, Maria Concepción García Otaduy, Roberta Diehl Rodriguez, Carlos Ernesto Garrido Salmon

**Affiliations:** ^1^ InBrain, Department of Physics, FFCLRP University of São Paulo Ribeirão Preto Brazil; ^2^ Graduate Program Neurology, FMRP University of São Paulo Ribeirão Preto Brazil; ^3^ Department of Physics, Huygens‐Kamerlingh Onnes Laboratory Leiden University Leiden the Netherlands; ^4^ LIM 44, InRad, FMUSP University of São Paulo São Paulo Brazil; ^5^ Department of Medical Imaging, Hematology and Clinical Oncology, FMRP University of São Paulo Ribeirão Preto Brazil

**Keywords:** brain, copper, EPR, ferritin, iron, paramagnetic ions

## Abstract

**Purpose:**

Paramagnetic ions are distributed throughout the human brain. The increased accumulation of these metals, such as iron and copper, can induce cellular death and the development of neurological diseases. Electron Paramagnetic Resonance (EPR) is a spectroscopic technique capable of detecting these ions in a given biological sample.

**Methods:**

Samples from 17 human brain structures of 8 ex vivo subjects were extracted, lyophilized, and triturated for EPR measurements at variable temperatures ranging from 193 to 293 K. Simulations were performed using the EasySpin toolbox to calculate qualitative parameters and the EPR absorption of high‐spin iron (Fe(III)), copper ion (Cu(II)), and ferritin (Ft) signals in all obtained EPR spectra.

**Results:**

The simulated parameters showed a considerable percentage variation relative to the input values, which resulted in spectral visual changes of each paramagnetic ion signal. The simulated EPR brain spectra demonstrated temperature dependence, with an increase in the amplitude of Fe(III), Cu(II), and Ft signals as the temperature decreased.

**Conclusions:**

The magnetic behavior of these paramagnetic species exhibited linearity with the inverse of temperature for the Cu(II) EPR absorption across all brain structures, while Fe(III) and Ft signals showed a nonlinear pattern in the EPR absorption, with heterogeneity among all brain regions and subjects.

## 1 Introduction

The human body has different paramagnetic trace elements that contribute to metabolic processes. Iron is an essential metal in the transportation of oxygen, and about 70% of the human body's iron (approximately 50 mg/kg body weight) is found in the hemoglobin [1]. At the cellular level, iron participates in the mitochondrial energy metabolism as an electron transporter or enzyme catalyst, and in the nucleus as a DNA repairer [2]. For copper, while in considerably lower concentrations in the whole body when compared to iron [3, 4], it is also present in the blood in the form of ceruloplasmin enzymes, which have the ability to oxidize Fe(II) into Fe(III). Furthermore, copper and iron have other metabolic crossroads in the human liver and brain [5], where both of them accumulate with aging [6, 7, 8, 9].

However, the imbalance of iron and copper increases the toxicity in the molecular environment and might cause cellular death. In the human body, this damage could induce liver diseases [10, 11] or neurological disorders, which some authors linked the development of Parkinson's disease [12, 13, 14], Alzheimer's disease [15, 16, 17] and Multiple Sclerosis [18, 19] with an increase of brain iron concentration.

In vivo metal quantification in the brain cannot be performed by an absolute method. Yet, Magnetic Resonance Imaging (MRI) techniques such as Quantitative Susceptibility Mapping (QSM) and R2* maps showed to be sensitive to the concentration of iron in the brain [20, 21, 22, 23, 24, 25]. QSM contrast is also age‐dependent in several gray matter regions [26, 27], while the changes of susceptibility caused by temperature variation are still under investigation [28, 29]. Unfortunately, it is difficult to create precise methodologies to evaluate this dependence for in vivo situations. Ex vivo studies using spectroscopic techniques might help assess the temperature effects on brain paramagnetic species.

Electron Paramagnetic Resonance (EPR) is a spectroscopic technique sensitive to paramagnetic ions in biological samples, represented by the signals in its spectrum as the first derivative of the sample's absorbance. The basic concepts of EPR are analogous to NMR, except that the excited spins are from unpaired electrons instead of the atomic nuclei spin. Quantitative information can be extracted from EPR spectra by the second integral of each paramagnetic ion signal, where the EPR absorption is proportional to the concentration or the magnetic susceptibility of the paramagnetic ion. Other quantitative information about each paramagnetic system can be obtained with dedicated simulations.

In post‐mortem human brain samples, EPR is able to detect both Fe(III) and Cu(II) paramagnetic ions contained in systems such as ferritin and neuromelanin, both iron‐based proteins [24, 30, 31, 32, 33]. Few studies carried out EPR measurements at variable temperature for post‐mortem brain samples, but only for few structures or subjects [32, 34, 35], leaving the magnetic behavior of these ions undefined for other brain structures among a larger size of subjects.

Other relevant EPR studies outside the human brain discuss mostly qualitative results of their spectra [36, 37, 38, 39, 40]. The qualitative discussion is, in general, limited to the presence or absence of a specific paramagnetic ion in the medium. Quantitative data is necessary to explore the differences in paramagnetic systems, such as the molecular environment and ion concentration in the whole brain.

The main objective of this study is to investigate the magnetic behavior of paramagnetic species as a function of temperature in post‐mortem human tissue from different brain regions. In the present study, the EPR data are analyzed to evaluate the effects on paramagnetic sites over a specific temperature range or across multiple brain regions and subjects. Furthermore, the Locus coeruleus (LC) is a brain structure that has rarely been investigated by EPR across different subjects, as previous studies tend to combine the LC tissue from multiple subjects into a pooled sample [30, 41]. The dataset of this study comprises a wide variety of brain structures obtained from multiple ex vivo subjects, which are condensed into an interpretable form to facilitate quantitative comparisons under different conditions. This approach provides insights into the physical and biological stability of certain paramagnetic systems and may serve as a basis for evaluating the impact of neurological disorders on these centers in future studies.

## 2 Methods

### 2.1 Human Brain Samples

**FIGURE 1:**
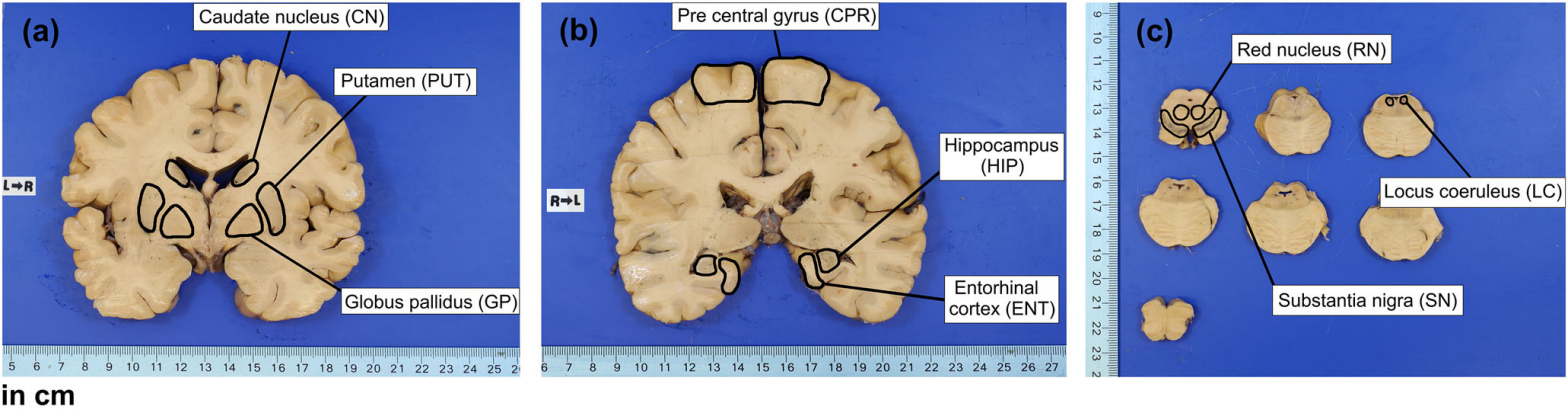
Coronal slices from brain and axial slices from the brainstem of fixed brain from a representative individual with the selected areas. (a) Coronal brain slice with Caudate nucleus (CN), Putamen (PUT) and Globus pallidus (GP); (b) coronal brain slice with Pre‐central gyrus (CPR), Hippocampus (HIP) and Entorhinal cortex (ENT); (c) axial brainstem slices of the midbrain with the Red nucleus (RN) and Substantia nigra (SN) and pons with the Locus coeruleus (LC).

This study was approved by the local ethics committee. Samples from nine regions: Caudate nucleus (CN), Putamen (PUT), Globus pallidus (GP), Pre‐central gyrus (CPR), Hippocampus (HIP), Entorhinal cortex (ENT), Red nucleus (RN), Locus coeruleus (LC), and Substantia nigra (SN) (Figure 1) were extracted from the brain of 8 male donors with ages ranging from 50 to 80 years old (68.3 ± 11.9 years old) without clinical diagnosis of any neurodegenerative disease according to a post‐mortem interview performed by trained gerontologists with a knowledgeable informant. Brain tissue was fixed in a 4% buffered paraformaldehyde within 24 h of death. After fixation, the brain was cut in consecutive coronal slices, while the brainstem in axial slices. To avoid metal contamination, a specific ceramic blade was used. The extraction of the samples of brain regions of interest (Figure 1) was performed by the same experienced pathologist in coronal slices of the brain and in axial slices of the brainstem using a specific ceramic blade to avoid metal contamination. Each sample area was separated into individual Eppendorf tubes for right and left sides, with the exception of the locus coeruleus (LC). For the LC, both hemispheres were combined because its sample quantity was lower than 25 mg for all subjects. The brain samples in the Eppendorfs were lyophilized, removing approximately 70% of liquid content, and manually triturated into dry powder. No additional chemical treatment was carried out.

### 2.2 EPR Spectral Acquisition

Spectral acquisition was performed for 17 brain structures, resulting in 136 samples. The wet and dry mass of each sample was measured before all acquisitions. An X‐band (9 GHz) EPR spectrometer (JEOL) was used with the parameters listed in Table 1. The temperature was decreased from 293 to 193 K in steps of 20 K using liquid nitrogen. No absolute calibration of the *g*‐values was performed. The correction of the field for frequency differences was not applied, since the relative variation in the microwave frequency at each spectral acquisition was below 0.1%. Larger variations could lead to shifts in the magnetic field and, therefore, would require correction.

**TABLE 1:** EPR spectra acquisition parameters for all brain samples.

EPR parameter	
Power	2 mW
Frequency interval	9.150 ± 0.003 GHz
Modulation amplitude	1 mT
Modulation frequency	100 kHz
Acquisition time	4 min
Field sweep	50–550 mT

### 2.3 Spectral Simulation and Quantification

**FIGURE 2:**
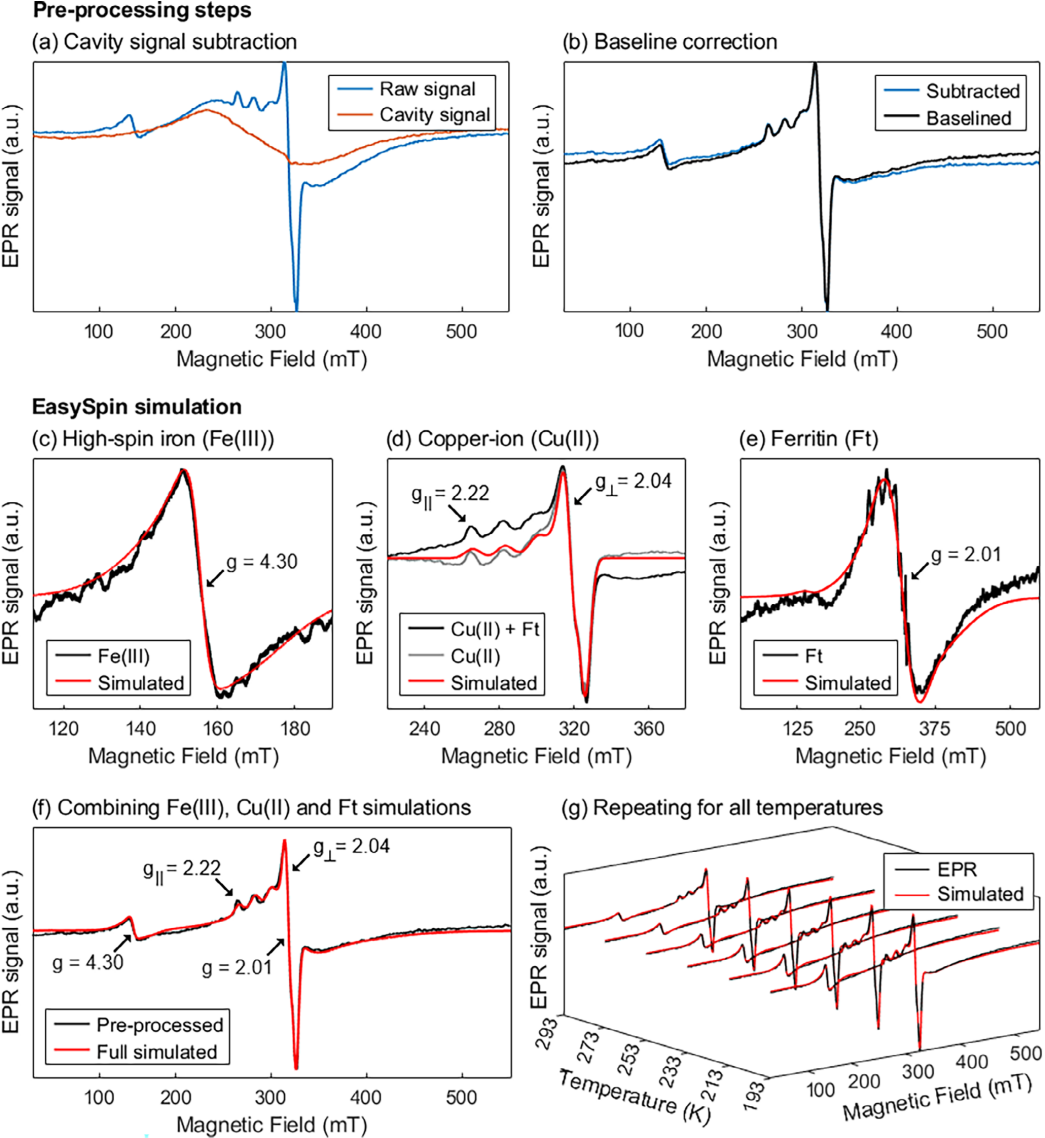
Simulation steps for EPR spectra at room temperature (293 K) of one representative subject's right putamen. First of all, the EPR raw signal is pre‐processed to (a) remove the cavity signal and to (b) correct the baseline displacement. Once the spectra are corrected, EasySpin simulation is individually performed for the (c) Fe(III), (d) Cu(II), and (e) Ft systems. After the simulations, (f) all three components (black arrows centered at the *g*‐values of the signals) are combined to build the fully simulated spectrum. Lastly, (g) these steps are repeated for the other temperatures.

The spectral simulation was performed by the EasySpin toolbox (version 6.0.2) in MATLAB (version R2022b) using the function pepper. A previous pipeline developed for brain EPR spectra simulations [32, 42] was used and improved for this study (Supporting Information document). All steps of pre‐processing and simulation are listed below; spectra are shown in Figure 2. In parentheses: the time needed to obtain a result of good quality in the simulation (typical desktop PC with 32 GB RAM and Intel Core i7‐10700F CPU).

1. Pre‐processing steps included the subtraction of EPR cavity signal from raw spectra (Figure 2a) and also baseline correction (1–5 min) resulting in pre‐processed raw spectra (Figure 2b). The method of baseline correction uses the 2nd integral over the full spectrum until zero slope is reached over the resulting plateau at the high‐field end of the spectrum [42].
2. Simulation of high‐spin iron (Fe(III)) signal centered at *g* = 4.30 from 50 mT to 220 mT field range (3–10 min, Figure 2c).
3. Subtraction of the Fe(III) simulated signal from raw spectra, resulting in copper‐ion + ferritin raw spectra (Figure 2d).
4. Smoothing using Fourier transform with 2nd order filtering was performed over full field range to isolate the copper‐ion (Cu(II)) signals from the ferritin (Ft) component (approximately 1 min, Figure 2d).
5. Simulation of Cu(II) signals between *g*_┴_ = 2.22 and *g*_‖_ = 2.04 from 220 to 370 mT field range (less than 3 min, Figure 2d).
6. Subtraction of the smoothed simulated Cu(II) signal from Cu(II) + Ft raw spectra, resulting in the Ft signal centered at *g* = 2.01 (Figure 2e).
7. Simulation of the Ft signal from Step 6. in the full field range (30 or more minutes, Figure 2e).
8. All the individual simulations were added up to build the full simulated EPR spectra (Figure 2f). Full simulated EPR spectra are then normalized by the sample dry mass used in the spectrometer.
9. The pre‐processing and simulation steps are then repeated for all temperatures (Figure 2g).
10. The absorption of each EPR signal is calculated as the second integral (from here on referred to as “EPR absorption”) along the field range at all temperatures, given that EPR signal is the first derivative of the sample's absorbance.

Some of the initial simulation parameters used in the EasySpin pipeline were based on previous studies [32, 33] and are listed in Table 2.

**TABLE 2:** Initial simulation parameters used in the EasySpin pipeline.

System	*S*	*g*_1_, *g*_2_	HStrain	*D* (MHz)	Weight
Ft (1)	10 [33]	2.010 (0.05) [33]	1509.3 (1000)	155.097 (50)	0.723 (0.15)
Ft (2)	10 [33]	2.010 (0.05) [33]	3931.9 (1000)	244.698 (50)	1.860 (0.15)

System	*S*	*g*_x_, *g*_y_, *g*_z_	gStrain	*D* (GHz)	*E* (GHz)
Fe(III)	5/2 [32]	1.818 (0.05)	0.222 (0.1) [32]	25.326 (5.0)	8.707 (0.5)
		1.955 (0.05)	0.042 (0.1) [32]		
		2.129 (0.05)	0.388 (0.1) [32]		

	*S*	*g*_‖_, *g*_┴_	gStrain	*A*_‖_, *A*_┴_ (MHz)
Cu(II)	1/2 [32]	2.219 (0.05)	0.031 (0.02)	38.892 (5.0)
		2.039 (0.05)	0.053 (0.02)	550.146 (20)

*Note*: Values in parenthesis represent the variation range for each parameter during the fitting of the signal. The *g*‐value defines the combination of magnetic field and microwave frequency of the resonance. Strain is the broadening of each g‐tensor component. The g‐tensor components reflect the symmetry of the signal: High‐spin iron (Fe(III)) has rhombic tensor (*g*_x_, *g*_y_, and *g*_z_ components differ), the copper‐ion (Cu(II)) signal has an axial symmetry (*g*_‖_ and *g*_┴_ components) and ferritin (Ft) is considered isotropic (single *g*‐value), with two different components (Ft(1) and Ft(2)) and weighed by the weight parameter. *D* and *E* are the zero‐field splitting constants and A is the anisotropic hyperfine tensor. *S* is the spin quantum number of each system.

### 2.4 Statistical Analysis

Signal‐to‐noise ratio (SNR) of all simulated spectra was calculated relative to the Fe(III) signal at *g* = 4.30 to qualitatively assess the influence of noise in the pre‐processing and simulation steps (Section 2.3).

Linear regression was performed to obtain the slope of the EPR absorption as a function of the inverse of the temperature. The calculation of the slope was weighted by the mean standard deviation; the mean slope of all brain structures was calculated only for the Cu(II) signal (Table 3).

**TABLE 3:** Slope of EPR absorption of the Cu(II) system from Figure 6 (mean slope α = 0.20 ± 0.01 K^−1^).

Brain region	CN	CPR	ENT	GP	HIP	LC	PUT	RN	SN
(1/K)	0.20	0.21	0.21	0.22	0.19	0.20	0.21	0.19	0.19

The percentage deviation of the simulated parameters from the EPR signal of each paramagnetic ion was calculated relative to the minimum simulated value:

(1) $$D\left(\%\right)=\frac{\left|X-X_{\min}\right|}{X_{\min}}100\%,$$

where X is the simulated value of the parameter of interest. Mean percentage deviation was calculated across all temperatures, subjects and brain regions (Figure 5).

## 3 Results

### 3.1 Simulated Spectra

The acquired EPR spectra at all temperatures had SNR between 5 and 50. EPR spectra at the lower end of the SNR range were not negatively affected by their noise during the pre‐processing and simulation steps performed for each paramagnetic ion's signal, as described in Section 2.3, and resulted in good simulations.

Fe(III) and Cu(II) signals were easily and quickly simulated in all EPR spectra. On the other hand, the Ft signal proved to be the most difficult to simulate, due to its various fitting parameters, larger field spread, and lower SNR values compared to Fe(III) and Cu(II) systems.

From all 136 available samples, 39 could not be simulated: six samples had abnormal signals close to the Fe(III) or Ft signals; five samples exhibited lyophilization problems and were discarded (EPR spectra were not acquired); and 28 had low dry mass after lyophilization. Thereby, the spectral simulation was performed on 97 brain samples at six temperatures (193 K). For all of these samples, 12 spectra were not acquired at a given temperature when the EPR spectrometer demonstrated instability or any error during the acquisition time. Overall, 570 spectra were fully simulated (Supporting Information in Figures S4–S11).

**FIGURE 3:**
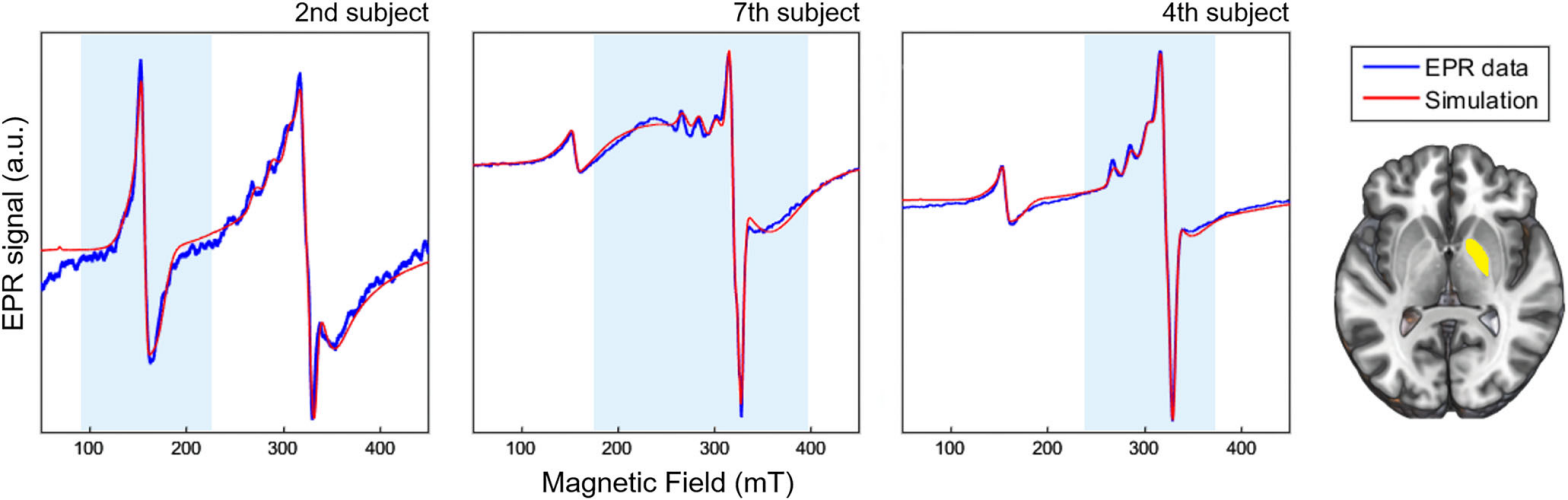
Differences between EPR spectra of three representative subjects for left globus pallidus (GP, highlighted in yellow) at the lowest temperature in the study (193 K). The second subject shows a strong contribution from the Fe(III) signal, while the seventh subject exhibits a larger Ft signal and the fourth subject displays a prominent contribution from the Cu(II) signal. For all these situations, the simulation agrees well with the measurement.

Differences in EPR spectra were observed between the subjects, with respect to the relative amplitude of the three simulated signals. Figure 3 shows EPR spectra for the same brain region and temperature of three different subjects in this study. In each case, the Fe(III), Ft, and Cu(II) signals had the highest amplitude along the field sweep compared to the two other contributions, respectively. These differences were also observed in different brain regions between the subjects and throughout the temperatures.

### 3.2 Simulated Parameters

After simulation of all EPR spectra, the simulated values of all parameters corresponding to each paramagnetic ion were obtained. The parameters that produced the most significant visual differences in the spectra were the g‐values across all three systems.

**FIGURE 4:**
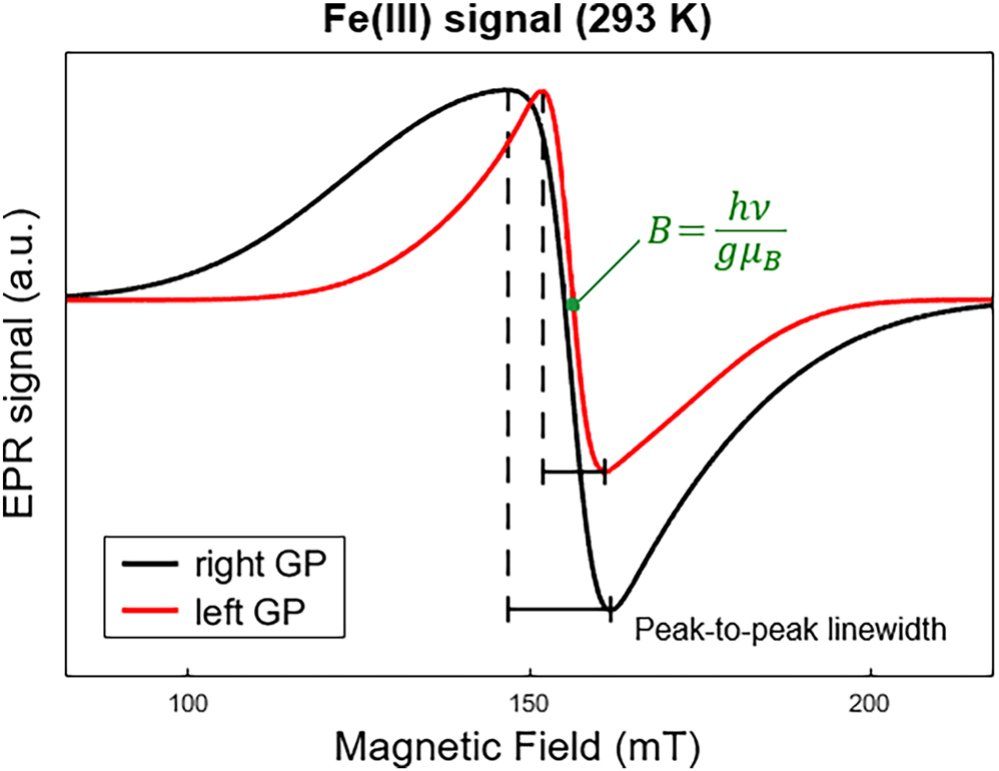
Simulated Fe(III) signal for right and left globus pallidus (GP) of the 7th subject. Green equation shows the applied magnetic field (B) in the zero‐crossing signal, where h is Planck constant, ν is the microwave frequency, $\mu_{B}$ is the Bohr magneton and g is the *g*‐value. Microwave frequencies are 9.148 GHz for the right GP and 9.149 GHz for the left GP. Note that dashed black lines show the peak‐to‐peak separation of both signals, visualizing the difference in the peak‐to‐peak linewidth of these two samples, where the lineshape can vary depending on the simulated *g*‐values or strain, for instance.

Figure 4 illustrates a practical example of these differences in the simulated EPR spectra from both sides of globus pallidus (GP) in room temperature (293 K) of a single subject. The peak‐to‐peak linewidth of right GP (Figure 4) was the result of a simulation with percentage variation of approximately 4% from the *g*_z_ input value (Table 2). In comparison to the left GP simulated spectrum (Figure 4), the percentage variations were less than 1% for *g*_z_. Small percentage variations in the g‐components of each system can cause considerable spectral visual changes.

**FIGURE 5:**
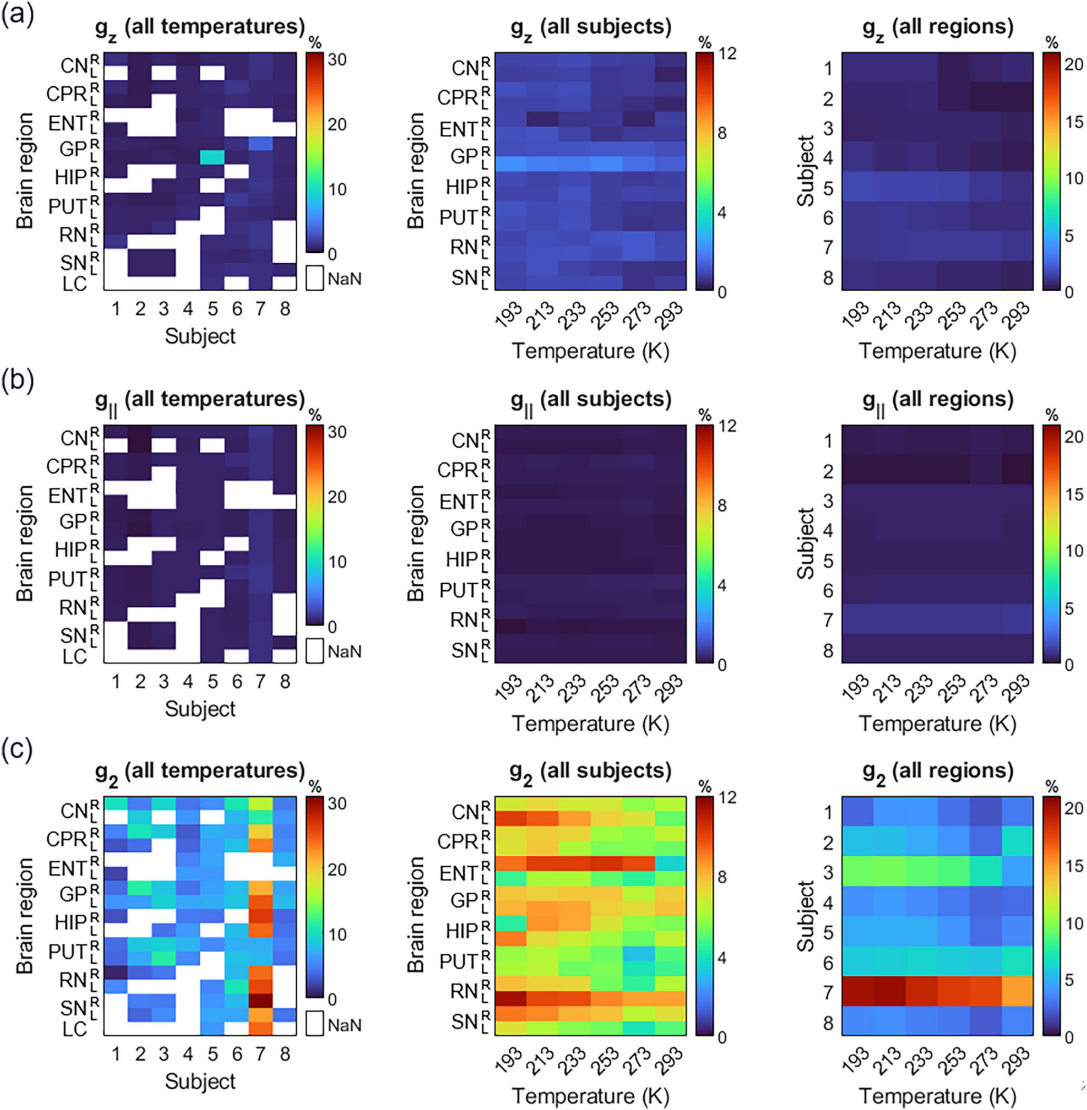
Mean percentage deviation of simulated g‐components: (a) g_z_‐component of Fe(III) signal, (b) g_||_‐component of Cu(II) signal and (c) g_2_‐component of Ft signal. The percentage deviations were calculated relative to the minimum simulated value of each parameter, as shown by Equation 1 (Section 2.4). Mean values were calculated across all temperatures, subjects and brain regions, respectively for left, middle and right heatmaps. The Y‐axis of left and middle heatmaps represents the right (R) and left (L) sides of all brain structures. The empty data (NaN) are due to missing brain samples for each subject in the present study.

The heatmaps presented in Figure 5 exhibit the mean percentage deviation of a single simulated g‐component of Fe(III), Cu(II), and Ft signals across all the temperatures, subjects, and brain regions. The bilateral LC was not represented in all three middle heatmaps because there were only two samples of this brain structure. These heatmaps show that the Fe(III) and Cu(II) ions remained considerably stable across these three variables (Figure 5a,b), with mean percentage variations of g‐value below 10% along the temperatures and not greater than 5% across subjects and brain regions. The left heatmap of the Fe(III) signal (Figure 5a) reveals two subjects with the highest variations in the mean g‐value of the GP structure; the same region is exemplified in Figure 4.

In relation to g‐components of the Ft signal (Figure 5c), the deviation values were significantly higher in percentage in comparison to both Fe(III) and Cu(II) signals. The 7th subject, for example, showed variations up to 20% in most of the brain regions across all temperatures in the left heatmap (Figure 5c), and also across the structures in the right heatmap (Figure 5c). High percentage variations were also observed for the other simulated parameters of Ft, and are exposed in the Supporting Information (Figure S3). These variations in the g‐component of the Ft signal also produced visual changes in its spectra for several brain regions of different subjects (Supporting Information in Figures S4–S11), analogous to the Fe(III) signal shown in Figure 4.

The acquired spectra of the simulated samples presented in Figure 4 were acquired at slightly different microwave frequencies (see caption of Figure 4). These differences in frequencies are not sufficiently large to explain the shift in line position, that is, *g*‐value.

### 3.3 Temperature Dependence of Paramagnetic Ions

**FIGURE 6:**
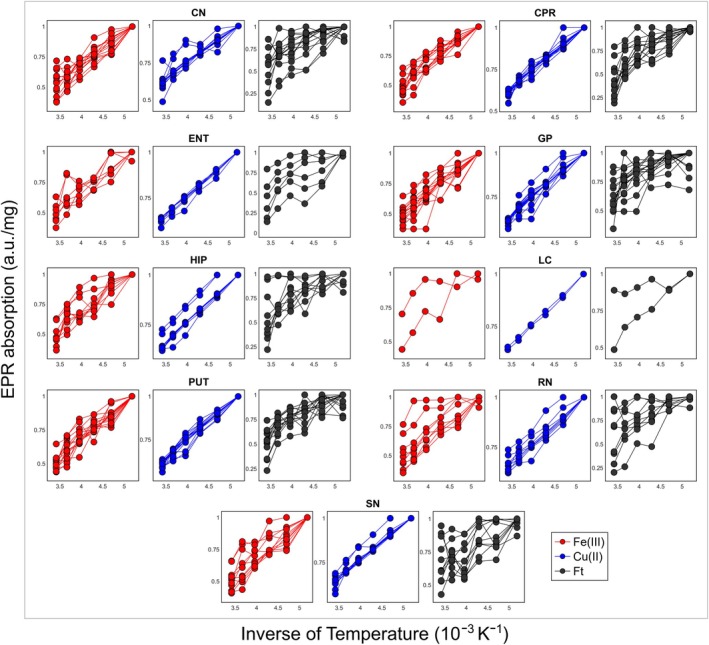
Normalized EPR absorption as a function of the inverse of temperature for several brain regions. Red, blue and black dots represent high‐spin iron (Fe(III)), copper‐ion (Cu(II)) and ferritin (Ft) signals, respectively. Each line is one ex vivo subject. Abbreviations are CN, Caudate nucleus; CPR, Pre‐central gyrus; ENT, Entorhinal cortex; GP, Globus pallidus; HIP, Hippocampus; LC, Locus coeruleus; PUT, Putamen; RN, Red nucleus, and SN, Substantia nigra.

The EPR absorptions calculated by the second integral were obtained for all temperatures in the range of 193 K for the Fe(III), Cu(II), and Ft signals from all simulated spectra. In general, the EPR absorptions increased as the temperature was lowered. These values were normalized to the maximum amplitude of the signal for each subject and represented as a function of the inverse of temperature in the same range, as shown in Figure 6. The normalized EPR absorption plotted as a function of the inverse of temperature is referred to as the Curie‐plot in the following.

Since the Cu(II) signal displayed a linear behavior of EPR absorption with the inverse of temperature, the slope of the curve of this paramagnetic ion was obtained for each brain structure (Table 3).

**FIGURE 7:**
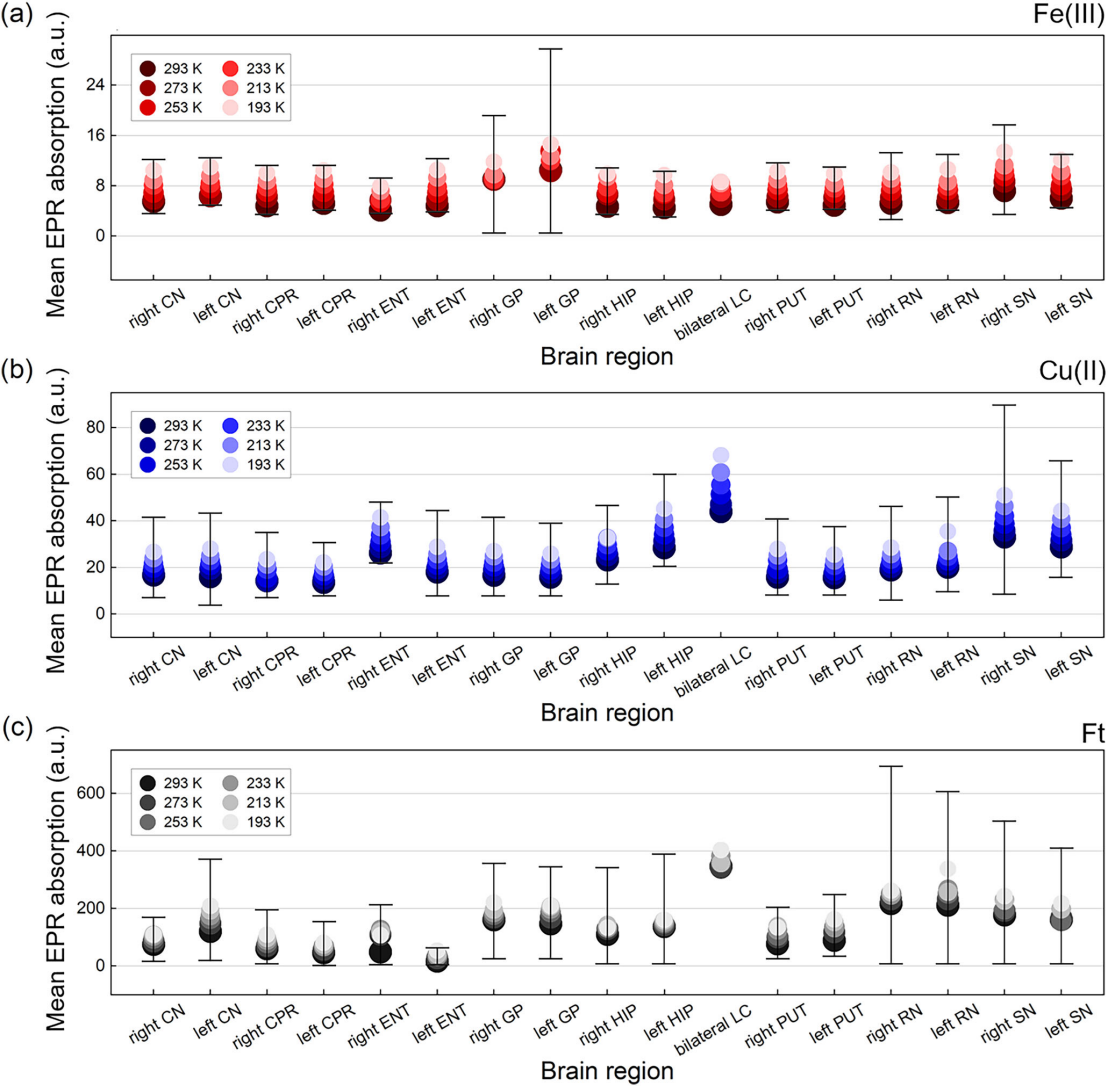
Mean EPR absorption of (a) Fe(III), (b) Cu(II) and (c) Ft sites from left and right sides of all brain structures, including the bilateral LC. The EPR absorption values are divided by 10^4^. The error bars represent the standard deviation interval from each calculated mean EPR absorption at each temperature. Lower temperatures are represented by lighter colors at each graph, as shown by their own legend.

The mean and standard deviation values across all subjects of the Fe(III), Cu(II), and Ft absorptions from all brain regions at all temperatures are shown in Figure 7. The right and left sides of the SN and GP had the highest values in the Fe(III) signal (Figure 7). For the Cu(II) signal, the LC and both sides of the SN had the highest mean EPR absorptions, while the GP, LC, RN, and SN demonstrated the highest EPR absorptions in the Ft signal (Figure 7). We observed significant mean deviations from some brain regions, such as the GP for the Fe(III) signals, SN for the Cu(II) signal, and the RN and SN for the Ft signal (Figure 7). These large standard deviation intervals occurred due to one representative subject that exhibited high EPR absorptions for all paramagnetic ions in these regions. The standard deviation intervals of the bilateral LC were not represented in Figure 7 because, in the present study, there were only two samples of this structure.

## 4 Discussion

This study aimed to investigate the temperature‐dependent properties of paramagnetic species in human brain tissue using EPR measurements at variable temperatures. The magnetic behavior of nine gray matter structures in the ex vivo human brain was assessed.

In contrast to elemental analysis techniques such as Inductively Coupled Plasma Mass Spectrometry (ICP‐MS), the EPR method allows for the identification of specific paramagnetic species. For example, in the case of iron, EPR can distinguish between isolated Fe(III) ions and ferritin‐bound iron. This advantage, however, comes with two notable limitations. First, diamagnetic or EPR‐silent species, such as Fe(II), are not detected. Second, the magnitude of the EPR absorption signal cannot be directly converted into absolute concentrations of these species (Figures 6 and 7); only relative amounts can be determined. Consequently, a complete characterization of brain samples would benefit from the inclusion of complementary analytical methods, such as ICP‐MS. Previous studies have combined EPR data from post‐mortem brain tissue with element concentrations obtained by ICP‐MS [24] and have correlated both measurements with quantitative MRI data [24, 25].

The percentage variation of all brain EPR spectral parameters with temperature is poorly explored in literature. In the present study, the percentage variations in the simulated parameters, as explained in Figure 5 and in the Supporting Information (Figures S1–S3), did not compromise the quality of each simulation, nor negatively affect the EPR absorption calculation from the Fe(III), Cu(II), and Ft contributions.

In the present study, we develop a new approach to present EPR results of a large number of samples in a compact form that is designed to give a quick overview of overall spectral properties. It also enables tracing differences in EPR parameters in a large number of brain regions and subjects. The heatmaps shown in Figure 5, and the temperature dependence plot in Figure 7, are illustrations of this. Our approach consists of two steps: (i) streamlining simulations of several paramagnetic centers in brain material following [32, 33, 36, 42] and (ii) representing the results as the variations in representative parameters. The resulting representations, for example, Figure 5, can condense a large amount of data to discover patterns that otherwise would go unnoticed. This made it possible to analyze and represent 570 simulated spectra in the present study. These heatmaps (Figure 5) contain information about the stability of the paramagnetic ion signal along all the simulations and across the temperature range.

The GP spectra shown in Figure 4 correspond to the g_z_‐component mean‐percentage variation found for a single subject in the left heatmap (Figure 5a). While these left heatmaps of g‐components represent the physical behavior of the Fe(III), Cu(II), and Ft signals, once the mean values were calculated across all temperatures, the middle and right heatmaps show the biological differences between different brain structures and subjects in all three paramagnetic ions.

These results (Figure 5) explain that Fe(III) and Cu(II) ions are physically stable at the temperatures studied, and biologically stable for different subjects and brain regions, while Ft does not present such stability. The lack of stability of this protein may suggest that there is a variety of Ft in different biological systems. However, it may also indicate that the proposed model for the Ft signal simulation is not sufficiently accurate. Recent studies addressed some difficulties that arise during the pre‐processing and simulation of this signal, while proposing some ways to reduce possible distortions in the Ft spectra [33, 42]. However, since these approaches were applied in the present study, it is unlikely that such distortions significantly affected the results.

The magnetic behavior of Fe(III), Cu(II), and Ft for each brain structure as a function of the inverse of temperature is seen in Figure 6. In the temperature range from 193 K to 293 K used in this study, Cu(II) (blue lines) exhibited a linear paramagnetic behavior for all structures, while Fe(III) and Ft (in red and black lines, respectively) did not show such linearity. The linear behavior of Cu(II) in this temperature range can be explained by Curie's classical law of magnetic susceptibility, given by χ = C/T, where C is a constant and T is the temperature. The non‐linearity observed in the EPR absorptions from both Fe(III) and Ft signals with 1/T suggests that these ions have additional contributions to their susceptibility in addition to the classical Curie paramagnetism [32, 43, 44].

Furthermore, the EPR absorption behavior of both Fe(III) and Ft with the inverse of temperature was heterogeneous across different brain structures. Also, the dispersion of the slopes of all Fe(III) and Ft curves (Figure 6) reflects the heterogeneity in the magnetism of these species between the subjects.

Studies analyzing the Curie‐plots for different brain structures are rare in the literature, but our results are consistent with the findings of Otsuka et al. [32]. In that study, the temperature range was considerably broader (from 5 to 300 K) and included only samples from the GP, SN, and LC of a single ex vivo subject. In our study, GP, SN, and LC also did not show a linear dependence of the EPR absorption with the inverse of temperature for the Fe(III) and Ft signals, as reported by Otsuka et al. [32].

In the temperature range from 193 to 293 K, same as used in the present study, Otsuka et al. observed that the EPR absorptions of Cu(II) from GP, SN and LC structures reached a reduced slope in comparison with the remainder of the temperature range [32]. The slope found in the present study (Table 3) for Cu(II) is similar to what was found by Otsuka et al. [32]. Yet, our mean slope of α = 0.20 ± 0.01 K^−1^ (Table 3) shows that all brain structures have the same linear behavior of Cu(II)'s EPR absorption as function of 1/T in the range from 193 K to 293 K, as the standard deviation represented only 4.9% of the absolute mean value.

Additionally, in Otsuka et al. the EPR absorptions of GP, SN and LC structures were fitted using the Curie–Weiss equation for Fe(III) and Cu(II) signals and a combination of Curie–Weiss and exponential Van‐Vleck equations for the Ft signal [32]. Unlike the classical Curie law equation, the Curie–Weiss formula depends on the Curie temperature ($T_{C}$), a point above which a ferromagnetic material loses its magnetic properties [44], while the Van‐Vleck formula consists of a sum of multiple exponentials, with their exponents proportional to 1/T [32, 43]. In addition to the linear regression performed for the Cu(II) signal to evaluate the classical Curie paramagnetism, none of these other equations were fitted to the absorption curves of Fe(III) or Ft in the present study, as this approach would not be meaningful given the temperature range used.

Especially for Ft in the human brain, the behavior of its EPR absorption over a broader temperature range from 5 to 300 K appears similar across these three brain structures, but exhibits different profiles depending on the temperature regime. Overall, from room temperature to 150 K, paramagnetic behavior was observed [32], as in the present study. Upon further lowering the temperature, the EPR absorption reached a plateau and then decreased with decreasing temperature [32].

This type of behavior is expected for Ft. Bossoni et al., who conducted similar EPR analyses of Ft isolated from human liver in a temperature range from 5 to 210 K [33], and found the same type of curve as Otsuka et al. observed for brain samples [32]. The maximum EPR absorption of liver Ft was approximately at 120 K, which is referred to as “blocking temperature”, below which antiferromagnetic behavior, that is, decreasing signal intensity with decreasing temperature, is observed [33]. Unfortunately, the present study is limited by a restricted temperature range from 193 to 293 K. This prevented us from reaching temperatures below 150 K to detect the blocking temperature of Ft in all brain structures. Further EPR studies using wider temperature ranges will be able to determine the blocking temperatures in several post‐mortem brain regions and between ex vivo subjects.

Furthermore, another biomolecule capable of storing Fe(III) ions is neuromelanin (NM) [45, 46]. NM is an iron‐based compound found throughout the human brain [41], but mainly concentrated in SN and LC dopaminergic neurons. In EPR spectra, NM organic radicals can be detected in brain samples with a specific chemical treatment at low temperatures. This chemical treatment was first carried out by Zecca et al. and repeated in several studies with the goal of characterizing NM in brain samples [30, 35, 47, 48].

Aime et al. and Lopiano et al. conducted studies with Q‐band (35 GHz) and X‐band (9 GHz) EPR of NM samples extracted from control and Parkinson's disease subjects [34, 35] at temperatures from 4 K to room temperature. For the 9 GHz EPR, Aime et al. and Lopiano et al. detected a signal of the NM organic radical around 330 mT at *g* = 2.0 [34, 35]. This signal has also been detected at the same position in other studies using similar methodologies [30, 41]. Aime et al. and Lopiano et al. [34, 35] also show that this radical species is difficult to detect in the presence of the paramagnetic metal ions naturally occurring in the SN and LC regions. They show that a chemical treatment to remove such paramagnetic metal ions enhances the radical signal. Therefore, it is expected that the contribution of this signal is weak, as observed in the spectra shown here.

In addition, the strong contribution from Cu(II) and Ft components partly masks the radical signal. Other factors are the measurement conditions, such as the large modulation amplitude and relatively high microwave power. Future studies assessing the NM organic radicals in EPR spectra of brain samples should be performed with experimental parameters adapted to the organic radicals to elucidate the magnetic behavior of this signal as a function of temperature.

The EPR absorption of each paramagnetic ion for all brain regions is shown in Figure 7. The LC was the brain structure with the highest mean value of the Cu(II) signal (Figure 7). This result is consistent with what is reported in the literature, as the LC is a copper‐rich human brain region [41, 49, 50]. For Fe(III), both the right and left sides of GP and SN exhibited the highest mean values (Figure 7). Some studies that evaluated the iron accumulation in the post‐mortem human brain also found high concentrations of this metal in these regions [9, 41, 51, 52], corroborating the results.

In contrast, the Ft signal showed large fluctuations in both mean and standard deviation values between different brain regions (Figure 7). These standard deviation values indicate that the Ft concentration is highly heterogeneous between the subjects of the present study in specific brain regions, such as the left and right sides of RN and SN (Figure 7). It should be pointed out that some of these fluctuations arise from the low SNR of the Ft spectra from several structures, which increases the variability in both mean and standard deviation values. Otsuka et al. carried out EPR measurements to quantify Ft in several brain regions and found large differences in the Ft from different subjects [24]. In that study, both sides of GP, RN, and SN had the highest mean values of EPR absorption [24], as found for the same regions in Figure 7. However, Otsuka et al. did not find high mean values of EPR absorption for the Ft in the bilateral LC [24], whereas the present study (Figure 7) shows the opposite. This difference might be due to the number of subjects, as the present study had only two LC samples, while Otsuka et al. had 13 samples for this same structure [24].

The present findings demonstrate the potential of EPR measurements to expand the quantitative analysis of changes in paramagnetic centers across multiple post‐mortem brain structures. Although the structural and biological implications of the observed temperature dependence remain to be clarified, the results provide a valuable basis for future investigations aiming to relate such effects to pathological factors. In this context, subsequent studies may explore the association of EPR results to specific pathological aspects of neurodegenerative diseases. Establishing the behavior of these paramagnetic centers for different subjects with no previous diagnosis in life of neurodegenerative disease is an essential first step, ensuring a reliable reference for later interpretation of the pathological effects in future studies.

## 5 Conclusions

The magnetic behavior and ion concentration of different paramagnetic species in the ex vivo human brain is heterogeneous between different brain structures and subjects. The EPR absorption of Cu(II) is linear as a function of the inverse of temperature, suggesting a Curie paramagnetic behavior, while both Fe(III) and ferritin's EPR absorptions do not follow a linear pattern, indicating additional contributions to the susceptibility apart from the Curie paramagnetism. The approach provides a method to visualize and compare EPR parameters across multiple brain regions and subjects, enabling the tracking of the changes observed here in larger statistical groups.

## Conflicts of Interest

The authors declare no conflicts of interest.

## Supporting information


**Figure S1.** Mean percentage deviation of Fe(III) simulated parameters (*g*_*x*_, *g*_*y*_, *g*_*z*_, *Strain*_*x*_, *Strain*_*y*_, *Strain*_*z*_, D and E) across temperatures, subjects and brain regions. Each heatmap shows the deviation averaged over one dimension (temperature, subject or region). Color scale represents the deviation magnitude in percent. These maps illustrate the relative variability of each parameter across experimental conditions, showing generally low inter‐temperature dependence, with higher variability observed for the strain components.
**Figure S2.** Mean percentage deviation of Cu(II) simulated parameters (*g*_*||*_, g_⊥_, *Strain*_||_, *Strain*_⊥_, *A*_||_, and *A*_⊥_) across temperatures, subjects and brain regions. Each heatmap shows the deviation averaged over one dimension (temperature, subject or region). Color scale represents the deviation magnitude in percent. These maps highlight the relative stability of Cu(II) simulated parameters across experimental conditions, with generally low variability across subjects and regions, and slightly higher deviations observed for the strain and anisotropy‐related parameters along temperatures.
**Figure S3.** Mean percentage deviation of Ft simulated parameters (*g*_1_, *g*_2_, *Strain*_1_, *Strain*_2_, *D*_1_, *D*_2_, *Weight*_1_ and *Weight*_2_) across temperatures, subjects and brain regions. Each heatmap shows the deviation averaged over one dimension (temperature, subject or region). Color scale represents the deviation magnitude in percent. These maps demonstrate that Ft simulated parameters across experimental conditions were generally higher in deviation in comparison to Fe(III) and Cu(II) simulated parameters, especially for the strain components and zero‐field splitting parameters.
**Figure S4.** EPR spectra of Fe(III), Cu(II) and Ft signals ranging from 193 to 293 K from all brain regions of the 1st ex vivo subject. Each set of panels displays the experimental spectrum (black line) and the individual simulated contributions at each temperature, as indicated in the legend below. This figure illustrates the paramagnetic behavior of these three species as the temperature decreases, with an increase of its signal amplitude.
**Figure S5.** EPR spectra of Fe(III), Cu(II) and Ft signals ranging from 193 to 293 K from all brain regions of the 2nd ex vivo subject. Each set of panels displays the experimental spectrum (black line) and the individual simulated contributions at each temperature, as indicated in the legend below. This figure illustrates the paramagnetic behavior of these three species as the temperature decreases, with an increase of its signal amplitude.
**Figure S6.** EPR spectra of Fe(III), Cu(II) and Ft signals ranging from 193 to 293 K from all brain regions of the 3rd ex vivo subject. Each set of panels displays the experimental spectrum (black line) and the individual simulated contributions at each temperature, as indicated in the legend below. This figure illustrates the paramagnetic behavior of these three species as the temperature decreases, with an increase of its signal amplitude.
**Figure S7.** EPR spectra of Fe(III), Cu(II) and Ft signals ranging from 193 to 293 K from all brain regions of the 4th ex vivo subject. Each set of panels displays the experimental spectrum (black line) and the individual simulated contributions at each temperature, as indicated in the legend below. This figure illustrates the paramagnetic behavior of these three species as the temperature decreases, with an increase of its signal amplitude.
**Figure S8.** EPR spectra of Fe(III), Cu(II) and Ft signals ranging from 193 to 293 K from all brain regions of the 5th ex vivo subject. Each set of panels displays the experimental spectrum (black line) and the individual simulated contributions at each temperature, as indicated in the legend below. This figure illustrates the paramagnetic behavior of these three species as the temperature decreases, with an increase of its signal amplitude.
**Figure S9.** EPR spectra of Fe(III), Cu(II) and Ft signals ranging from 193 to 293 K from all brain regions of the 6th ex vivo subject. Each set of panels displays the experimental spectrum (black line) and the individual simulated contributions at each temperature, as indicated in the legend below. This figure illustrates the paramagnetic behavior of these three species as the temperature decreases, with an increase of its signal amplitude.
**Figure S10.** EPR spectra of Fe(III), Cu(II) and Ft signals ranging from 193 to 293 K from all brain regions of the 7th ex vivo subject. Each set of panels displays the experimental spectrum (black line) and the individual simulated contributions at each temperature, as indicated in the legend below. This figure illustrates the paramagnetic behavior of these three species as the temperature decreases, with an increase of its signal amplitude.
**Figure S11.** EPR spectra of Fe(III), Cu(II) and Ft signals ranging from 193 to 293 K from all brain regions of the 8th ex vivo subject. Each set of panels displays the experimental spectrum (black line) and the individual simulated contributions at each temperature, as indicated in the legend below. This figure illustrates the paramagnetic behavior of these three species as the temperature decreases, with an increase of its signal amplitude.

## Data Availability

The data that support the findings of this study are available from the corresponding author upon reasonable request.
