# Supplementary material for: Temperature Dependence of Paramagnetic Species in the Human Brain Tissue: An X‐Band EPR Study
Source: Magn Reson Med. 2025 Dec 12;95(5):3031–43. doi: 10.1002/mrm.70222 (PMC12962191; doi:10.1002/mrm.70222)
Supplement: Supplementary file 1 — Figure S1. Mean percentage deviation of Fe(III) simulated parameters (g x , g y , g z , Strain x , Strain y , Strain z , D and E) across temperatures, subjects and brain regions. Each heatmap shows the deviation averaged over one dimension (temperature, subject or region). Color scale represents the deviation magnitude in percent. These maps illustrate the relative variability of each parameter across experimental conditions, showing generally low inter‐temperature dependence, with higher variability observed for the strain components. Figure S2. Mean percentage deviation of Cu(II) simulated parameters (g || , g⊥, Strain ||, Strain ⊥, A ||, and A ⊥) across temperatures, subjects and brain regions. Each heatmap shows the deviation averaged over one dimension (temperature, subject or region). Color scale represents the deviation magnitude in percent. These maps highlight the relative stability of Cu(II) simulated parameters across experimental conditions, with generally low variability across subjects and regions, and slightly higher deviations observed for the strain and anisotropy‐related parameters along temperatures. Figure S3. Mean percentage deviation of Ft simulated parameters (g 1, g 2, Strain 1, Strain 2, D 1, D 2, Weight 1 and Weight 2) across temperatures, subjects and brain regions. Each heatmap shows the deviation averaged over one dimension (temperature, subject or region). Color scale represents the deviation magnitude in percent. These maps demonstrate that Ft simulated parameters across experimental conditions were generally higher in deviation in comparison to Fe(III) and Cu(II) simulated parameters, especially for the strain components and zero‐field splitting parameters. Figure S4. EPR spectra of Fe(III), Cu(II) and Ft signals ranging from 193 to 293 K from all brain regions of the 1st ex vivo subject. Each set of panels displays the experimental spectrum (black line) and the individual simulated contributions at each temperature, as indicated in t [file MRM-95-3031-s001.pdf]

Supplementary Information

A. Processing pipeline for EPR spectra of brain sample (MATLAB code):
https://github.com/andreavanzine/EPR-brain-simulation.git

B. Simulated parameters

High-spin iron (Fe(III))

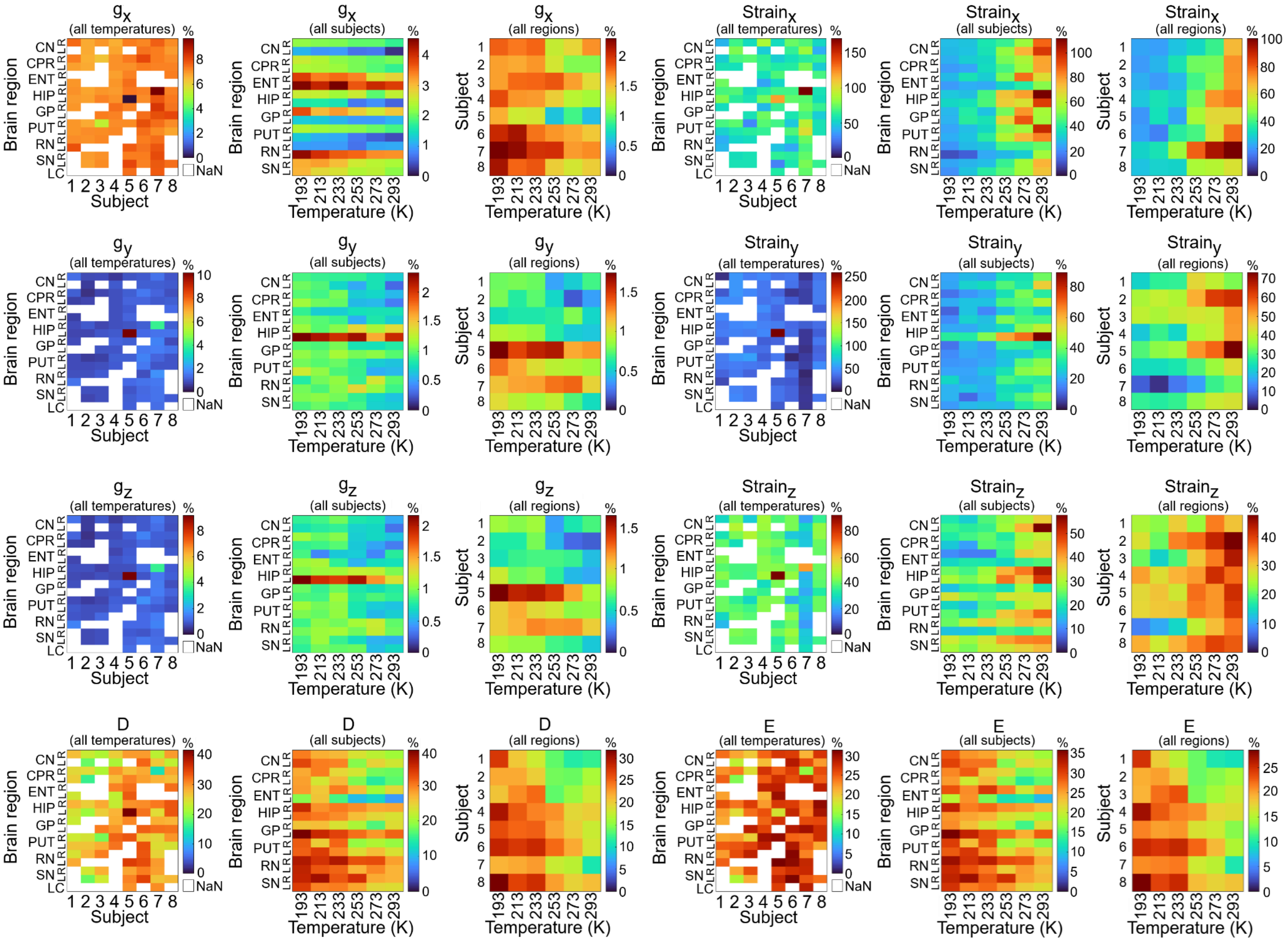

Figure S1. Mean percentage deviation of Fe(III) simulated parameters (g_x, g_y, g_z, $Strain_x$, $Strain_y$, $Strain_z$, D and E) across temperatures, subjects and brain regions. Each heatmap shows the deviation averaged over one dimension (temperature, subject or region). Color scale represents the deviation magnitude in percent. These maps illustrate the relative variability of each parameter across experimental conditions, showing generally low inter-temperature dependence, with higher variability observed for the strain components.

Copper-ion (Cu(II))

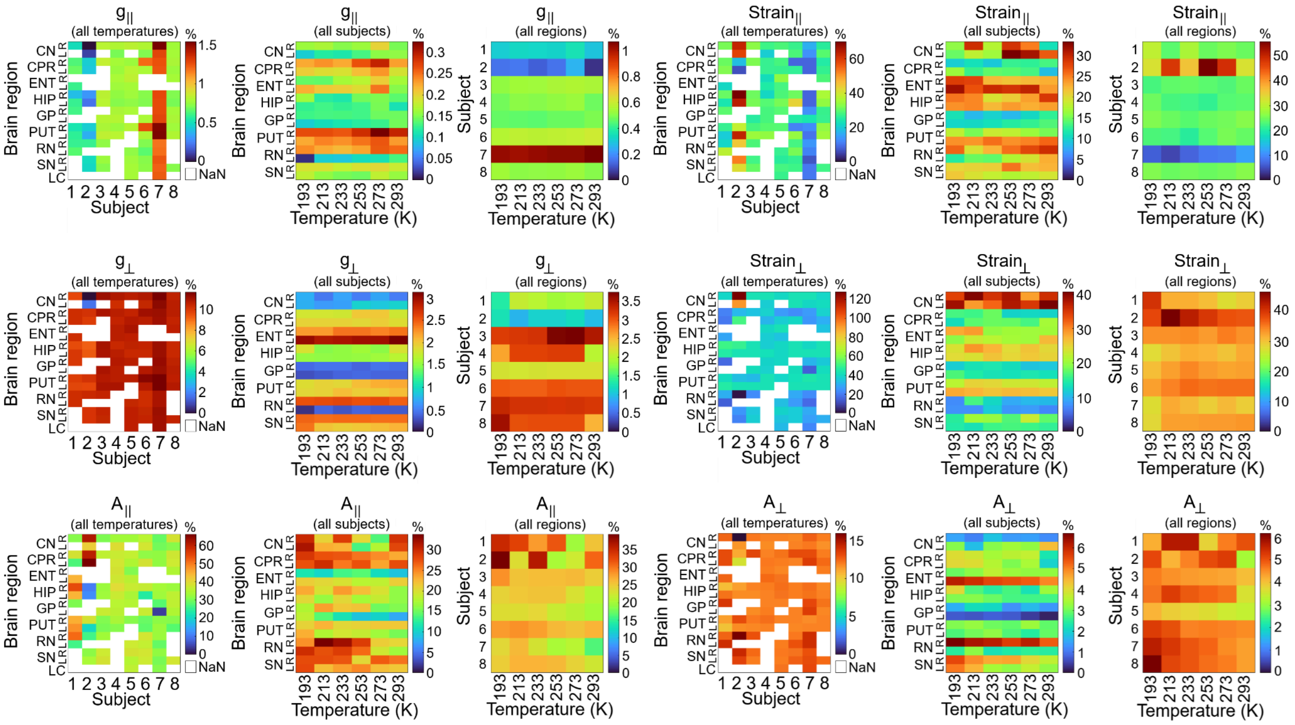

Figure S2. Mean percentage deviation of Cu(II) simulated parameters ($g_{||}$, $g_{\perp}$, $Strain_{||}$, $Strain_{\perp}$, $A_{||}$ and $A_{\perp}$) across temperatures, subjects and brain regions. Each heatmap shows the deviation averaged over one dimension (temperature, subject or region). Color scale represents the deviation magnitude in percent. These maps highlight the relative stability of Cu(II) simulated parameters across experimental conditions, with generally low variability across subjects and regions, and slightly higher deviations observed for the strain and anisotropy-related parameters along temperatures.

Ferritin (Ft)

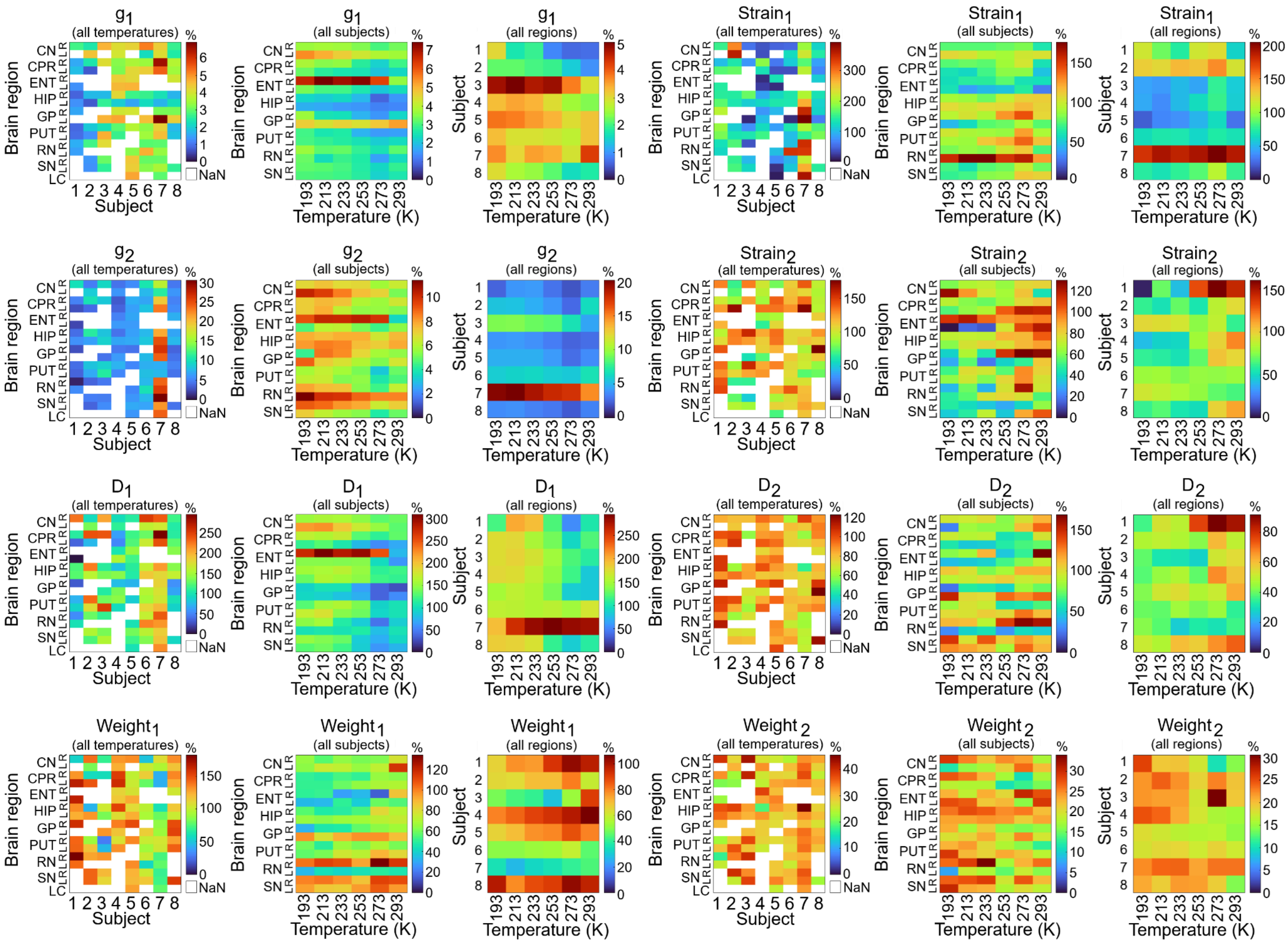

Figure S3. Mean percentage deviation of Ft simulated parameters (g_1, g_2, $Strain_1$, $Strain_2$, D_1, D_2, $Weight_1$ and $Weight_2$) across temperatures, subjects and brain regions. Each heatmap shows the deviation averaged over one dimension (temperature, subject or region). Color scale represents the deviation magnitude in percent. These maps demonstrate that Ft simulated parameters across experimental conditions were generally higher in deviation in comparison to Fe(III) and Cu(II) simulated parameters, especially for the strain components and zero-field splitting parameters.

C. Acquired and simulated EPR spectra

Subject 1

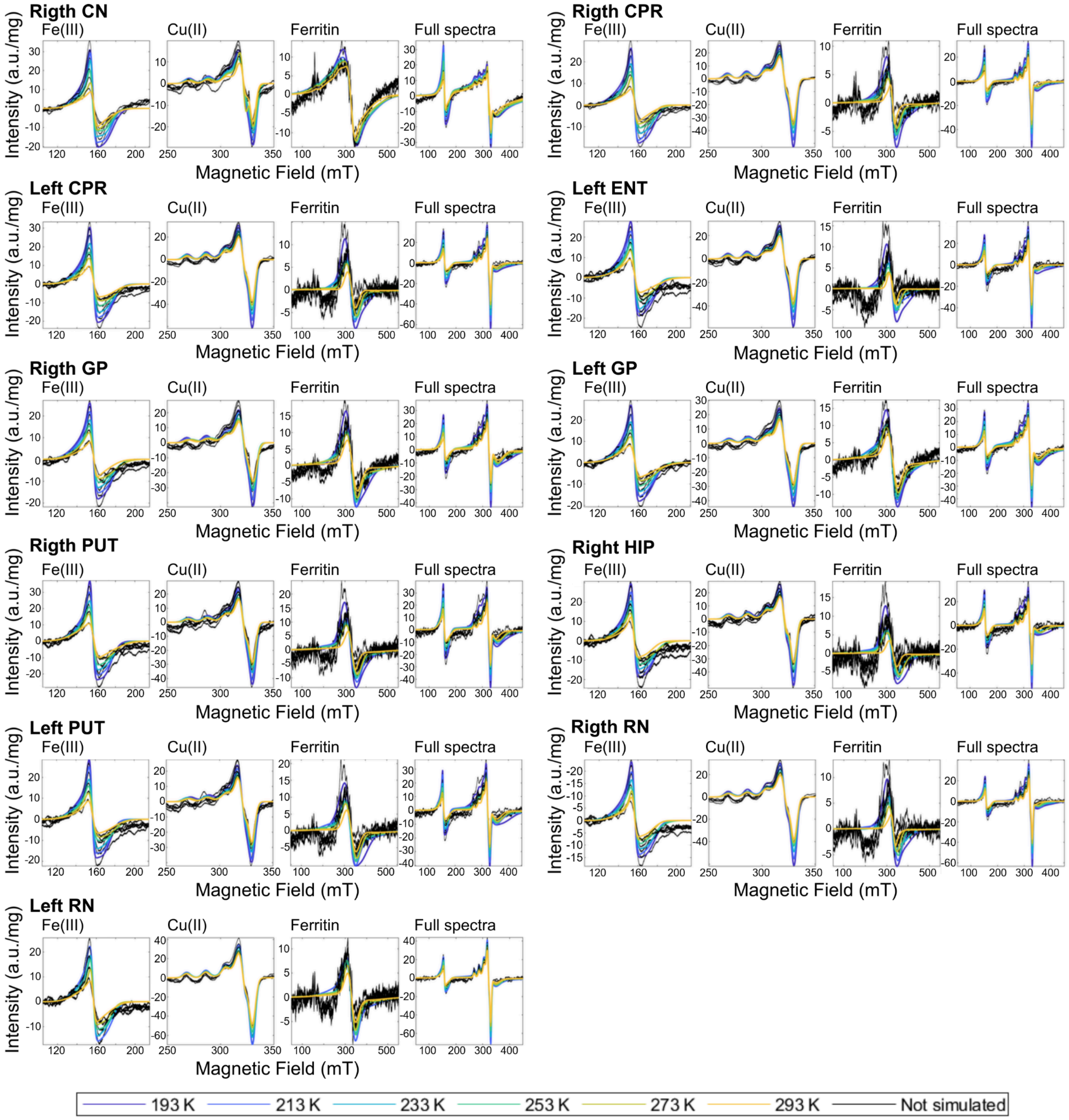

Figure S4. EPR spectra of Fe(III), Cu(II) and Ft signals ranging from 193 to 293 K from all brain regions of the 1st *ex-vivo* subject. Each set of panels displays the experimental spectrum (black line) and the individual simulated contributions at each temperature, as indicated in the legend below. This figure illustrates the paramagnetic behavior of these three species as the temperature decreases, with an increase of its signal amplitude.

Subject 2

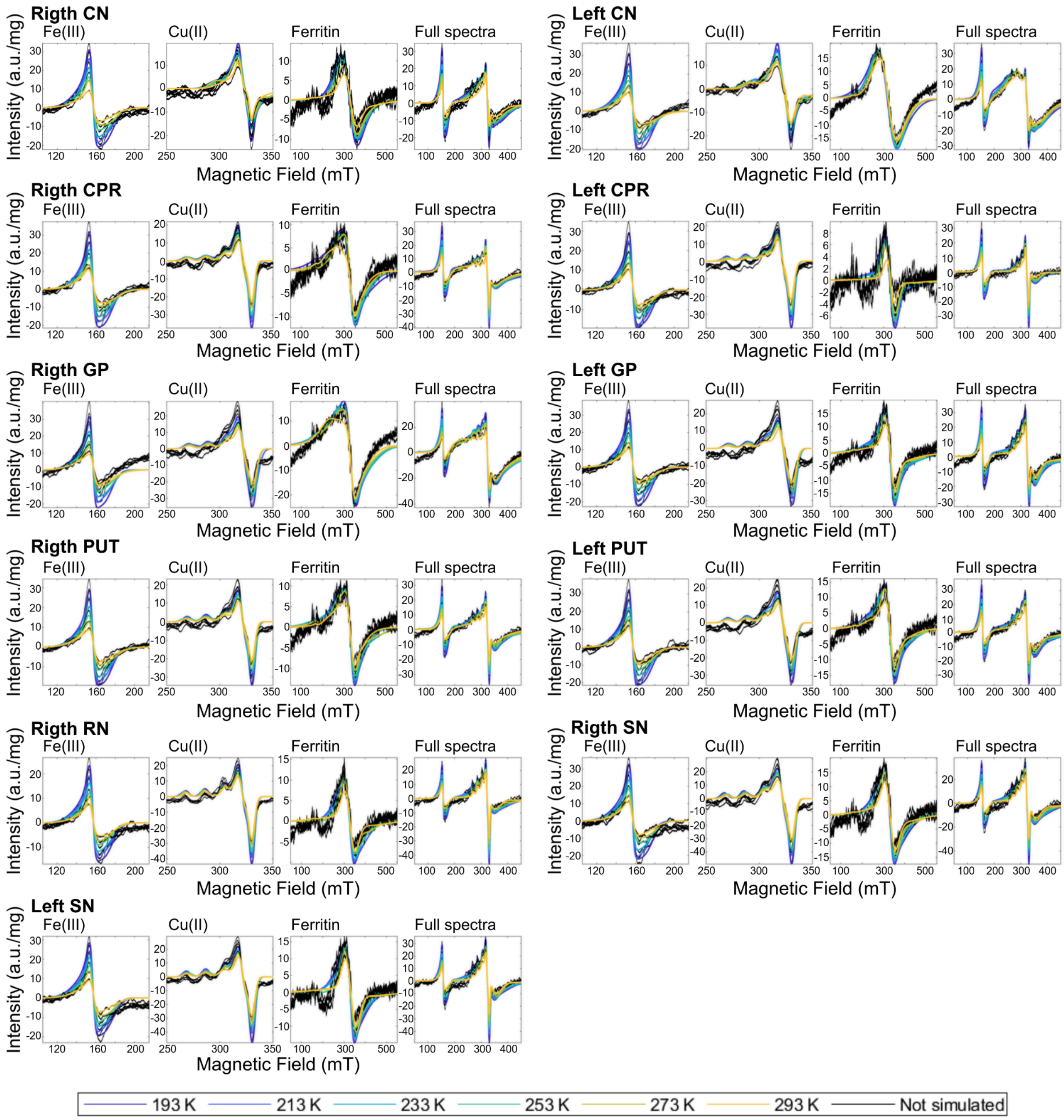

Figure S5. EPR spectra of Fe(III), Cu(II) and Ft signals ranging from 193 to 293 K from all brain regions of the 2nd *ex-vivo* subject. Each set of panels displays the experimental spectrum (black line) and the individual simulated contributions at each temperature, as indicated in the legend below. This figure illustrates the paramagnetic behavior of these three species as the temperature decreases, with an increase of its signal amplitude.

Subject 3

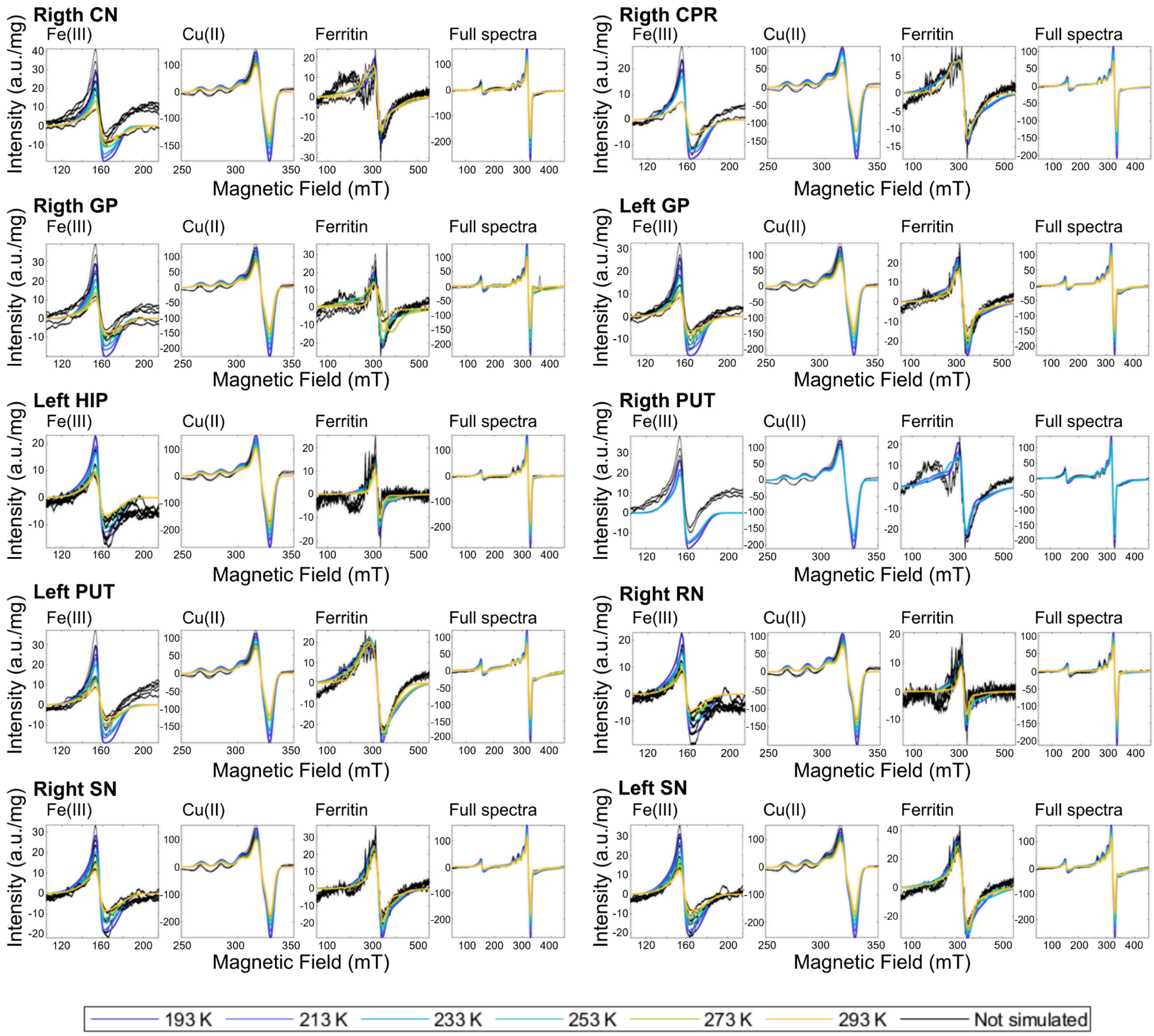

Figure S6. EPR spectra of Fe(III), Cu(II) and Ft signals ranging from 193 to 293 K from all brain regions of the 3rd *ex-vivo* subject. Each set of panels displays the experimental spectrum (black line) and the individual simulated contributions at each temperature, as indicated in the legend below. This figure illustrates the paramagnetic behavior of these three species as the temperature decreases, with an increase of its signal amplitude.

Subject 4

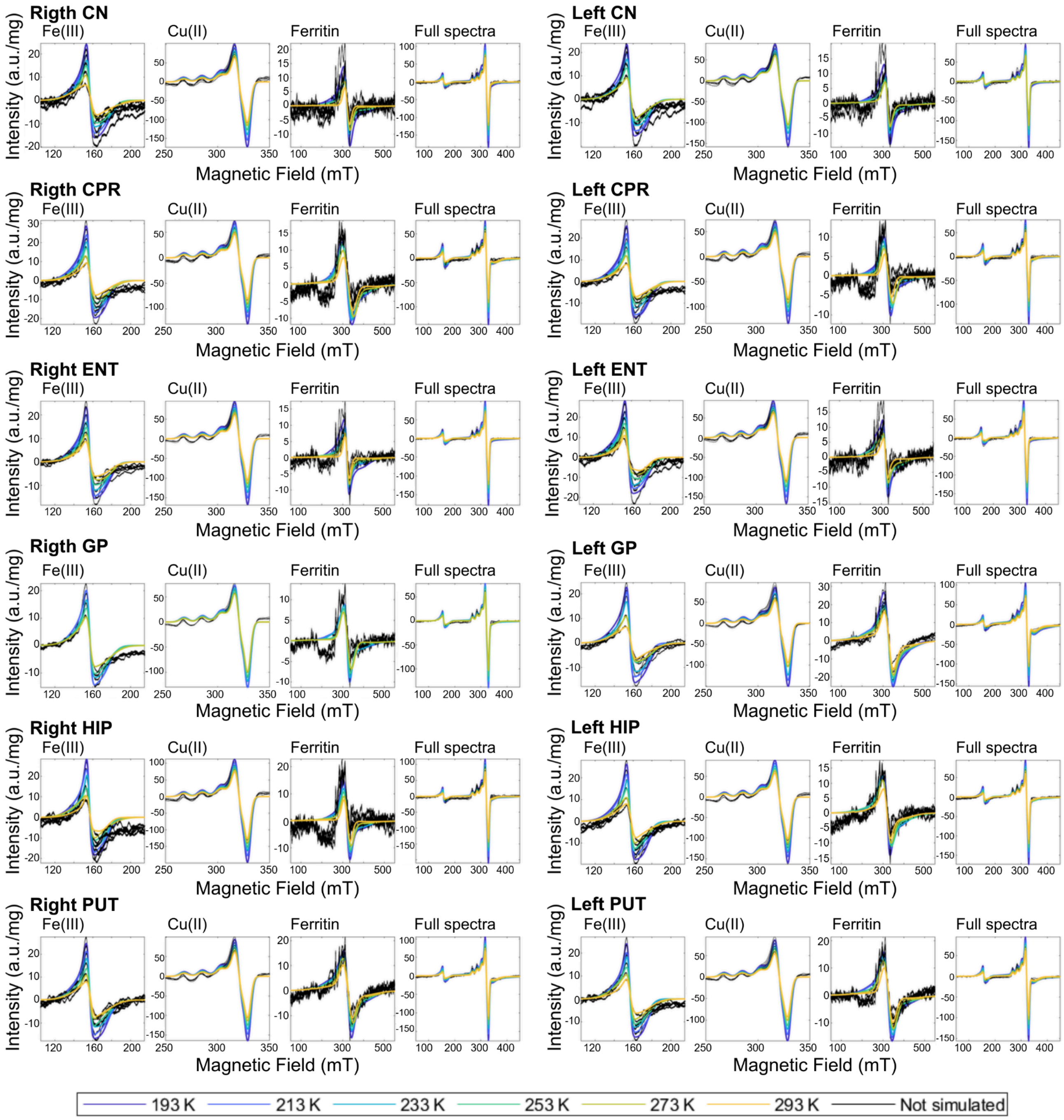

Figure S7. EPR spectra of Fe(III), Cu(II) and Ft signals ranging from 193 to 293 K from all brain regions of the 4th *ex-vivo* subject. Each set of panels displays the experimental spectrum (black line) and the individual simulated contributions at each temperature, as indicated in the legend below. This figure illustrates the paramagnetic behavior of these three species as the temperature decreases, with an increase of its signal amplitude.

Subject 5

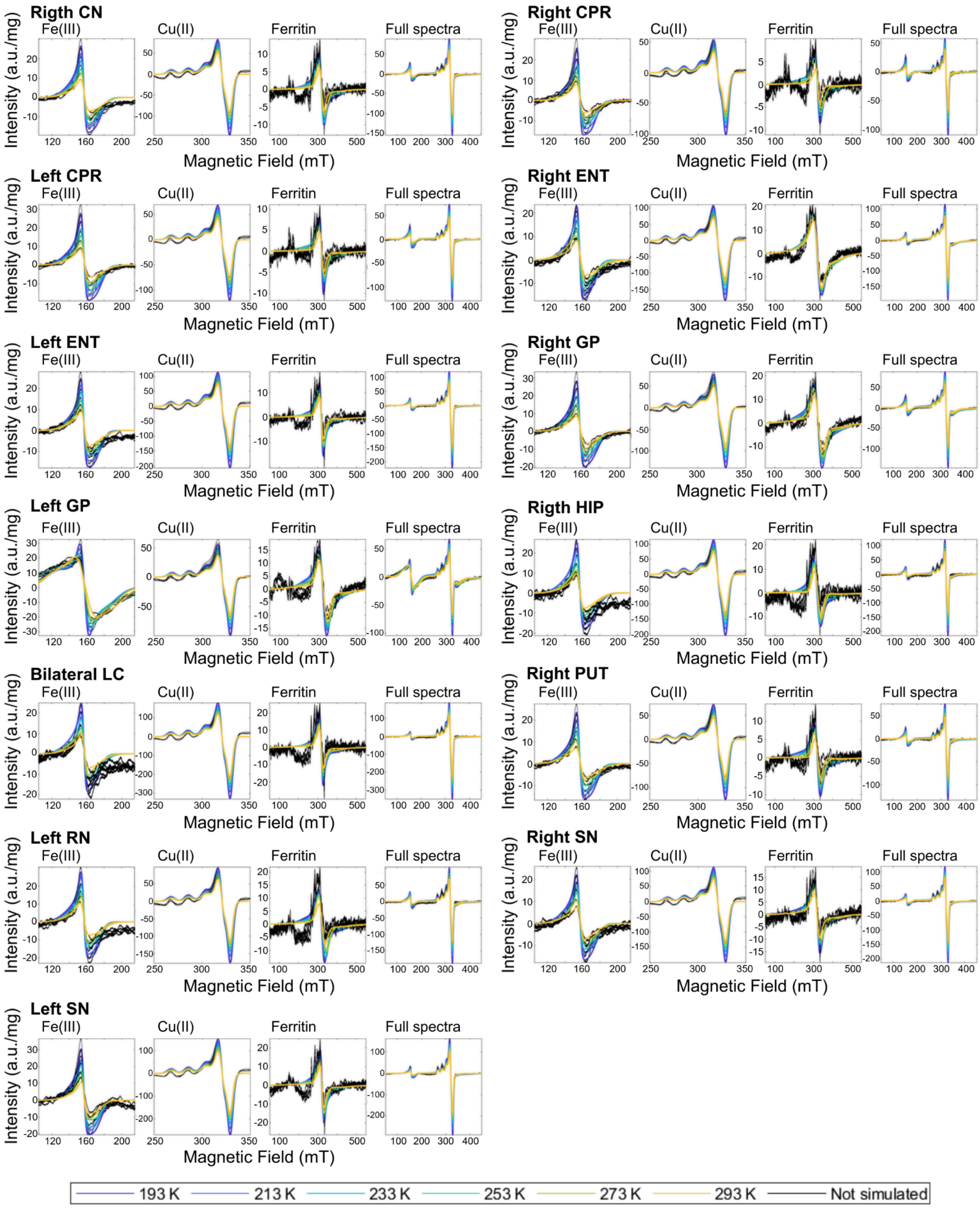

Figure S8. EPR spectra of Fe(III), Cu(II) and Ft signals ranging from 193 to 293 K from all brain regions of the 5th *ex-vivo* subject. Each set of panels displays the experimental spectrum (black line) and the individual simulated contributions at each temperature, as indicated in the legend below. This figure illustrates the paramagnetic behavior of these three species as the temperature decreases, with an increase of its signal amplitude.

Subject 6

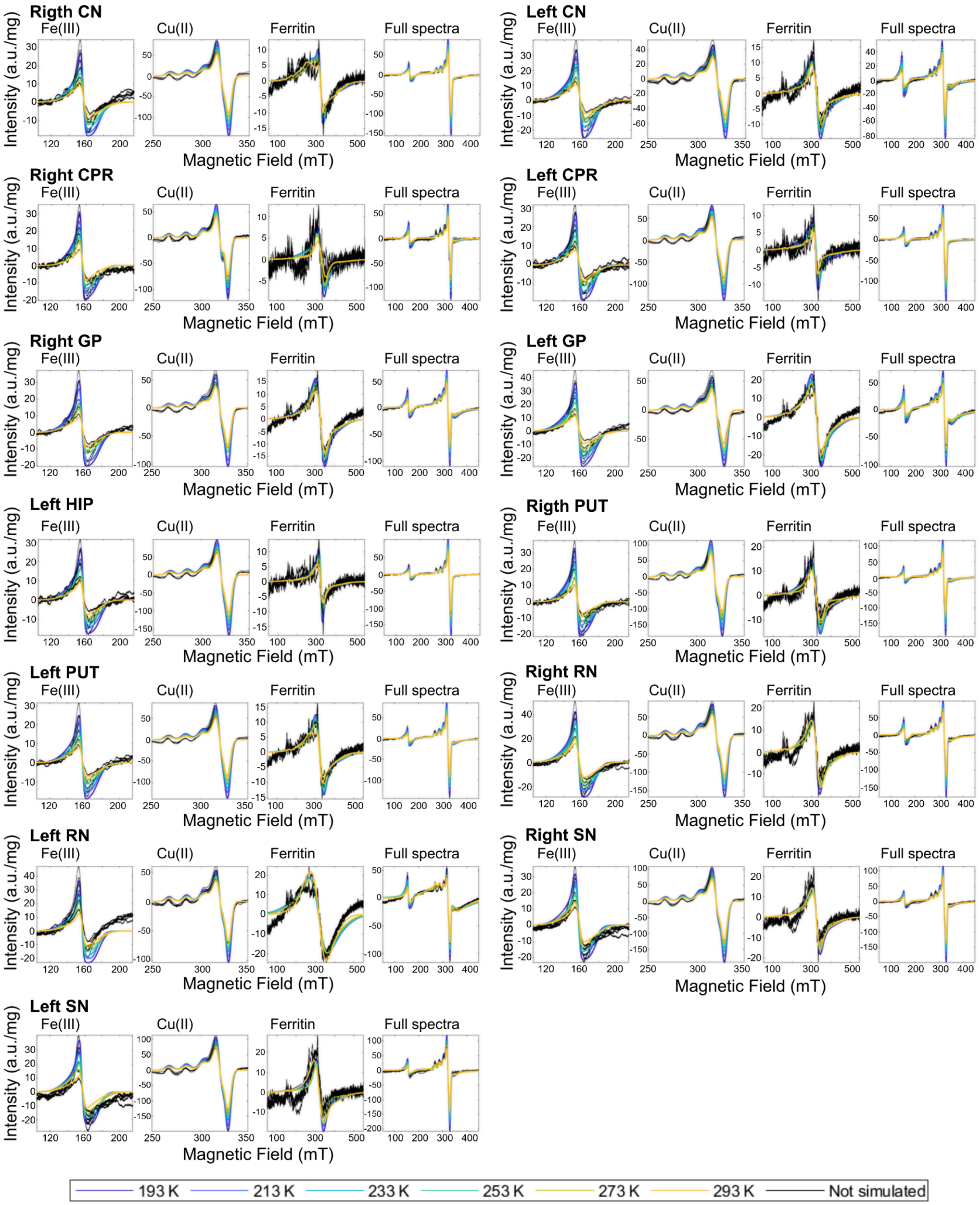

Figure S9. EPR spectra of Fe(III), Cu(II) and Ft signals ranging from 193 to 293 K from all brain regions of the 6th *ex-vivo* subject. Each set of panels displays the experimental spectrum (black line) and the individual simulated contributions at each temperature, as indicated in the legend below. This figure illustrates the paramagnetic behavior of these three species as the temperature decreases, with an increase of its signal amplitude.

Subject 7

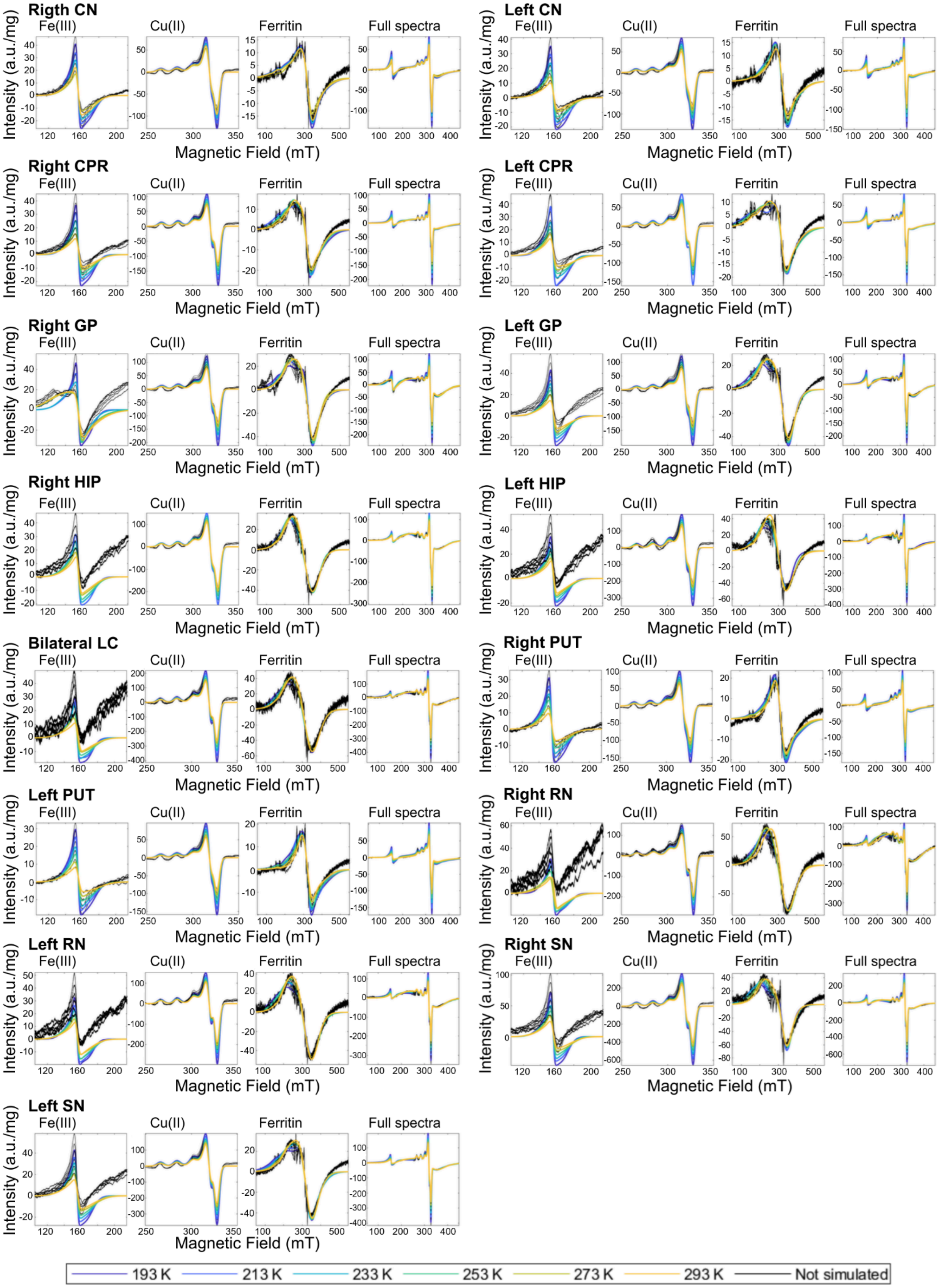

Figure S10. EPR spectra of Fe(III), Cu(II) and Ft signals ranging from 193 to 293 K from all brain regions of the 7th *ex-vivo* subject. Each set of panels displays the experimental

spectrum (black line) and the individual simulated contributions at each temperature, as indicated in the legend below. This figure illustrates the paramagnetic behavior of these three species as the temperature decreases, with an increase of its signal amplitude.

Subject 8

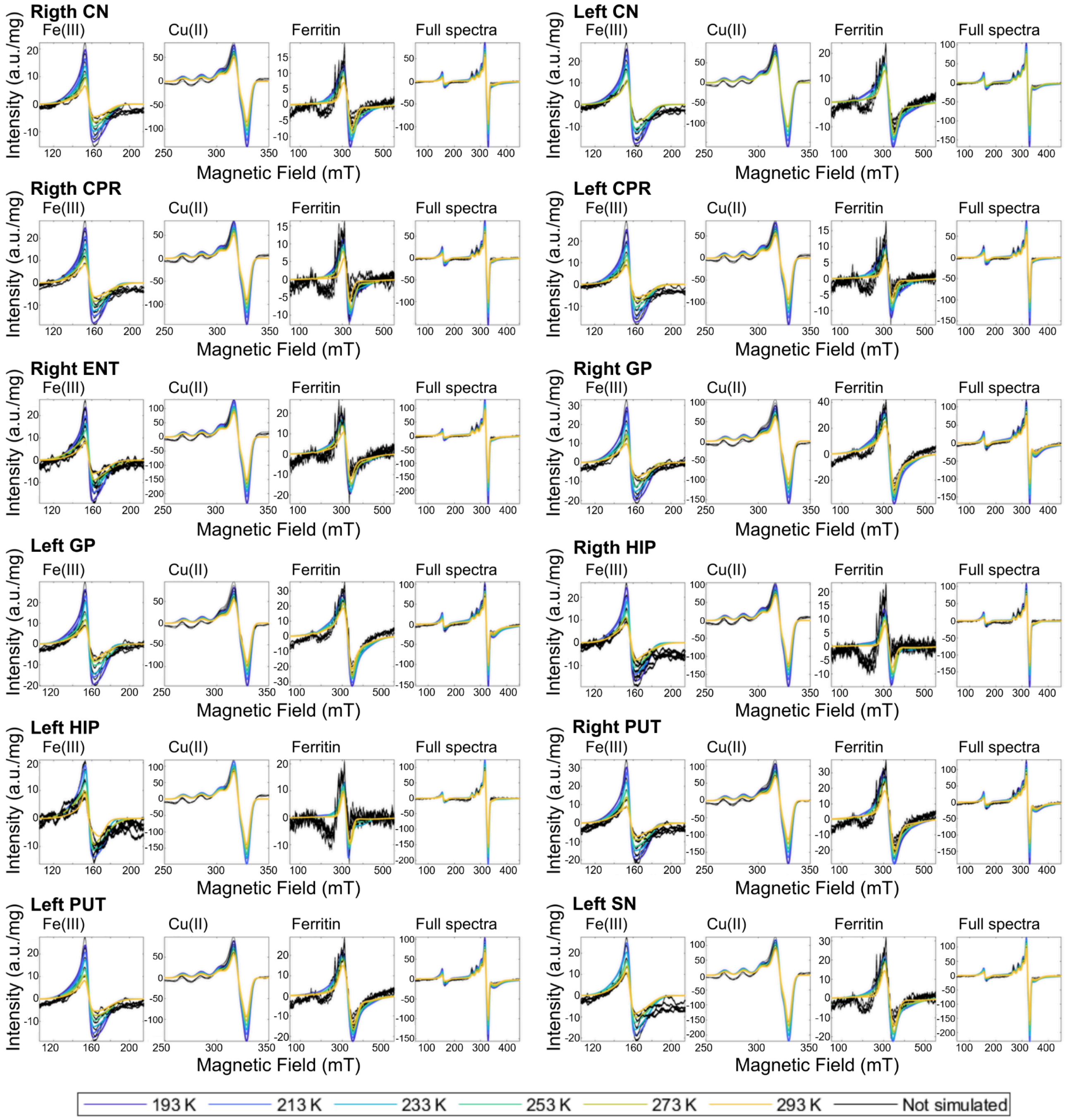

Figure S11. EPR spectra of Fe(III), Cu(II) and Ft signals ranging from 193 to 293 K from all brain regions of the 8th *ex-vivo* subject. Each set of panels displays the experimental spectrum (black line) and the individual simulated contributions at each temperature, as indicated in the legend below. This figure illustrates the paramagnetic behavior of these three species as the temperature decreases, with an increase of its signal amplitude.
